# New Books

**Published:** 2007-09

Alternative Pathways in Science and Industry: Activism, Innovation, and the Environment in an Era of Globalization

David Hess

Cambridge, MA:MIT Press, 2007. 260 pp. ISBN: 978-0-262-58272-8, $25

Assessment of the Continuing Operability of Chemical Agent Disposal Facilities and Equipment

Committee on Continuing Operability of Chemical Agent Disposal Facilities and Equipment, National Research Council

Washington, DC:National Academies Press, 2007. 78 pp. ISBN: 978-0-309-10351-0, $18

Bioinformatics: Experiments, Tools, Databases, and Algorithms

Orpita Bosu, Simminder Kaur Thukral

New York:Oxford University Press, 2007. 528 pp. ISBN: 978-0-19-567683-9, $49.95

Clearing the Air: The Health and Economic Damages of Air Pollution in China

Mun S. Ho, Chris P. Nielsen, eds.

Cambridge, MA:MIT Press, 2007. 392 pp. ISBN: 978-0-262-08358-4, $50

Climate Change: What It Means for Us, Our Children, and Our Grandchildren

Joseph F. C. DiMento, Pamela M. Doughman, eds.

Cambridge, MA:MIT Press, 2007. 232 pp. ISBN: 978-0-262-04241-3, $60

Ecology of Tidal Freshwater: Forested Wetlands of the Southeastern United States

William H. Conner, Thomas W. Doyle, Ken W. Krauss, eds.

New York:Springer, 2007. 506 pp. ISBN: 978-1-4020-5094-7, $239

Environmental Health Risk IV

C.A. Brebbia, ed.

Billerica, MA:WIT Press, 2007. 304 pp. ISBN: 978-184564-083-5, $185

Environmental Impacts of Wind-Energy Projects

Committee on Environmental Impacts of Wind Energy Projects, National Research Council

Washington, DC:National Academies Press, 2007. 346 pp. ISBN: 978-0-309-10830-0, $56

Fundamentals of Environmental Sampling and Analysis

Chunlong Zhang

Hoboken, NJ:John Wiley & Sons, 2007. 436 pp. ISBN: 978-0-471-71097-4, $99.95

Geostatistics for Environmental Scientists, 2nd ed.

Richard Webster, Margaret A. Oliver

Hoboken, NJ:John Wiley & Sons, 2007. 304 pp. ISBN: 978-0-470-02858-2, $120

Greenhouse Gas Sinks

D. Reay, N. Hewitt, K. Smith, J. Grace

New York:Oxford University Press, 2007. 448 pp. ISBN: 978-1-8459-3189-6, $160

Inclusion: The Politics of Difference in Medical Research

Steven Epstein

Chicago:University of Chicago Press, 2007. 424 pp. ISBN: 978-0-226-21309-5, $29.00

Markets and the Environment

Nathaniel O. Keohane, Sheila M. Olmstead

Washington, DC:Island Press, 2007. 288 pp. ISBN: 1-59726-047-9, $19.95

Methods and Tools for Drought Analysis and Management

Giuseppe Rossi, Teodoro Vega, Brunella Bonaccorso, eds.

New York:Springer, 2007. 418 pp. ISBN: 978-1-4020-5923-0, $199

Pollution of the Sea: Prevention and Compensation

Jürgen Basedow, Ulrich Magnus, eds.

New York:Springer, 2007. 183 pp. ISBN: 978-3-540-73395-9, $109

Public Health Advocacy and Tobacco Control: Making Smoking History

Simon Chapman

Oxford, UK:Blackwell, 2007. 336 pp. ISBN: 978-1-405-16163-3, $59.95

Risk Assessment for Environmental Health

Mark G. Robson, William A. Toscano, eds.

San Francisco:Jossey-Bass, 2007. 664 pp. ISBN: 978-0-7879-8319-2, $85

Sediment Dredging at Superfund Megasites: Assessing the Effectiveness

Committee on Sediment Dredging at Superfund Megasites, National Research Council

Washington, DC:National Academies Press, 2007. 236 pp. ISBN: 978-0-309-10973-4, $45

Sustainable Poverty Reduction in Less-Favoured Areas

R. Ruben, J. Pender, A. Kuyvenhaven

New York:Oxford University Press, 2007. 464 pp. ISBN: 978-1-8459-32-7, $170

Taking Action, Saving Lives: Our Duties to Protect Environmental and Public Health

Kristin Shrader-Frechette

New York:Oxford University Press, 2007. 320 pp. ISBN: 978-0-19-532546-1, $29.95

The Middle Path: Avoiding Environmental Catastrophe

Eric Lambin

Chicago:University of Chicago Press, 2007. 208 pp. ISBN: 978-0-226-46853-2, $25

The Unnatural History of the Sea

Callum Roberts

Washington, DC:Island Press, 2007. 464 pp. ISBN: 1-59726-102-5, $28

The WTO Agreement on Sanitary and Phytosanitary Measures: A Commentary

Joanne Scott

New York:Oxford University Press, 2007. 360 pp. ISBN: 978-0-19-927112-2, $140

